# Supplementary material for: An ethogram of acute pain behaviors in cats based on expert consensus
Source: PLoS One. 2023 Sep 28;18(9):e0292224. doi: 10.1371/journal.pone.0292224 (PMC10538801; doi:10.1371/journal.pone.0292224)
Supplement: S1 Table — (DOCX) [file pone.0292224.s001.docx]

# Supplementary Information S1

| **Categories** | **Behavior** | **Member 1** | **Member 2** | **Member 3** | **Member 4** | **Experts in agreement** | **I-CVI** |
| --- | --- | --- | --- | --- | --- | --- | --- |
| **Position in the cage** | Back (D) | 3 | 3 | 4 | 3 | 4 | 1 |
|  | Front (D) | 3 | 3 | 1 | 1 | 2 | 0.5 |
| **Exploratory behavior** | Withdrawing (F) (D) | 4 | 4 | 4 | 2 | 3 | 0.75 |
|  | Investigating (D) | 3 | 4 | 2 | 2 | 2 | 0.5 |
|  | No attention to the surroundings (D) | 3 | 4 | 4 | 3 | 4 | 1 |
| **Activity** | Restlessness (D) | 4 | 2 | 3 | 3 | 3 | 0.75 |
|  | Feign sleep (F) (D) | 4 | 3 | 4 | 4 | 4 | 1 |
|  | Grooming (D) | 3 | 4 | 3 | 3 | 4 | 1 |
|  | Stretching (F) | 3 | 4 | 1 | 4 | 3 | 0.75 |
|  | Scratching (F) | 2 | 4 | 2 | 2 | 1 | 0.25 |
|  | Tail twitch (F) | 3 | 2 | 2 | 2 | 1 | 0.25 |
|  | Attention to the wound (F) | 4 | 4 | 4 | 4 | 4 | 1 |
| **Posture & Body Position** | Sitting (D) | 3 | 4 | 1 | 2 | 2 | 0.5 |
|  | Crouched/Hunched up (D) | 4 | 4 | 4 | 3 | 4 | 1 |
|  | Lying dorsoventrally with pelvic limbs extended/contracted (F) (D) | 4 | 4 | 2 | 3 | 3 | 0.75 |
|  | Abnormal gait (F) | 3 | 3 | 3 | 3 | 4 | 1 |
|  | Non-weight bearing (F) | 4 | 4 | 4 | 2 | 3 | 0.75 |
| **Affective-emotional states** | Depressed (D) | 4 | 3 | 4 | 3 | 4 | 1 |
|  | Repelling (F) | 3 | 2 | 4 | 3 | 3 | 0.75 |
| **Vocalization** | Growling (F) | 3 | 2 | 3 | 3 | 3 | 0.75 |
|  | Hissing (F) | 3 | 2 | 3 | 3 | 3 | 0.75 |
| **Playing (with an object)** | Pawing (D) | 3 | 4 | 1 | 3 | 3 | 0.75 |
|  | Not pawing but interest to play/towards the object (D) | 3 | 2 | 1 | 1 | 1 | 0.25 |
|  | Not pawing/no interest towards the object (D) | 4 | 2 | 2 | 1 | 1 | 0.25 |
| **Feeding** | Eating (D) | 3 | 2 | 1 | 2 | 1 | 0.25 |
|  | Difficulty grasping or holding food (F) | 4 | 4 | 3 | 4 | 4 | 1 |
|  | Head shaking during feeding (F) | 4 | 4 | 3 | 3 | 4 | 1 |
|  | Not eating but interest in food (D) | 4 | 2 | 2 | 3 | 2 | 0.5 |
|  | Not eating/no interest in food (D) | 4 | 2 | 3 | 3 | 3 | 0.75 |
| **Post feeding** | Lip licking (F) | 3 | 2 | 3 | 2 | 2 | 0.5 |
|  | Grooming (D) | 4 | 3 | 3 | 1 | 3 | 0.75 |
| **Facial expressions or features** | Head shaking (not related to eating) (F) | 4 | 3 | 3 | 1 | 3 | 0.75 |
|  | Eye squinting (F) | 4 | 4 | 4 | 4 | 4 | 1 |
|  | Blepharospasm (F) | 4 | 4 | 3 | 3 | 4 | 1 |
|  | Lip licking (not related to eating) (F) | 2 | 2 | 3 | 2 | 1 | 0.25 |
|  | Lowered head position (D) | 3 | 4 | 4 | 3 | 4 | 1 |

(1) not relevant, (2) somewhat relevant, (3) quite relevant, or (4) highly relevant to the measured domain; I-CVI = Item Content Validity Index (No. of Agreement (per item)/No of Experts)
