# Supplementary material for: An ethogram of acute pain behaviors in cats based on expert consensus
Source: PLoS One. 2023 Sep 28;18(9):e0292224. doi: 10.1371/journal.pone.0292224 (PMC10538801; doi:10.1371/journal.pone.0292224)
Supplement: S1 File — (DOCX) [file pone.0292224.s002.docx]

# Supplementary Information S2

1. Strong WS. Through a glass darkly: Using behavior to assess pain. Semin Vet Med Surg Small Anim. 1997;12(2):61–74.

2. Cambridge AJ, Tobias KM, Newberry RC, Sarkar DK. Subjective and objective measurements of postoperative pain in cats. J Am Vet Med Assoc. 2000 Sep;217(5):685–90. Available from: http://avmajournals.avma.org/doi/abs/10.2460/javma.2000.217.685

3. Mathews KA. Pain assessment and general approach to management. Vet Clin North Am - Small Anim Pract [Internet]. 2000;30(4):729–55. Available from: http://dx.doi.org/10.1016/S0195-5616(08)70004-4

4. Patronek GJ. Assessment of claims ofshort- and long-term complications associated with onychectomy in cats. J Am Vet Med Assoc. 2001;219(7).

5. Cloutier S, Newberry RC, Cambridge AJ, Tobias KM. Behavioural signs of postoperative pain in cats following onychectomy or tenectomy surgery. Appl Anim Behav Sci. 2005;92(4):325–35.

6. Robertson SA, Taylor PM. Pain management in cats—past, present and future. Part 2. Treatment of pain—clinical pharmacology. J Feline Med Surg. 2004 Oct 25;6(5):321–33. Available from: http://journals.sagepub.com/doi/10.1016/j.jfms.2003.10.002

7. Robertson SA. Assessment and management of acute pain in cats. J Vet Emerg Crit Care. 2005;15(4):261–72.

8. Wilson D V., Pascoe PJ. Pain and analgesia following onychectomy in cats: A systematic review. Vet Anaesth Analg. 2016;43(1):5–17.

9. Flecknell P. Analgesia from a veterinary perspective. Br J Anaesth. 2008;101(1):121–4. Available from: http://dx.doi.org/10.1093/bja/aen087

10. Reid J, Nolan AM, Scott EM. Measuring pain in dogs and cats using structured behavioural observation. Vet J. 2018;236:72–9. Available from: https://doi.org/10.1016/j.tvjl.2018.04.013

11. Brondani JT, Luna LSP, Beier SL, Minto BW, Padovani CR. Analgesic efficacy of perioperative use of vedaprofen, tramadol or their combination in cats undergoing ovariohysterectomy. J Feline Med Surg. 2009 Jun 1;11(6):420–9. Available from: http://journals.sagepub.com/doi/10.1016/j.jfms.2008.10.002

12. Rütgen BC, Stüger HP, Holzmann A, Bubna-Littitz H, Skalicky M, Thalhammer JG. Postoperative pain-associated behaviour in cats undergoing ovariohysterectomy. Wien Tierarztl Monatsschr. 2011;98(5–6):119–25.

13. Downing s R. Pain management for veterinary palliative care and hospice patients. Vet Clin North Am - Small Anim Pract [Internet]. 2011;41(3):531–50. Available from: http://dx.doi.org/10.1016/j.cvsm.2011.03.010

14. Brondani JT, Luna S, Padovani CR. Assessing Acute Postoperative Pain in Cats. Am J Vet Res. 2011;72(2):174–83.

15. Brondani JT, Mama KR, Luna SPL, Wright BD, Niyom S, Ambrosio J, et al. Validation of the English version of the UNESP-Botucatu multidimensional composite pain scale for assessing postoperative pain in cats. BMC Vet Res. 2013 Dec 17;9(1):143. Available from: https://bmcvetres.biomedcentral.com/articles/10.1186/1746-6148-9-143

16. Brondani JT, Luna SPL, Minto BW, Santos BPR, Beier SL, Matsubara LM, et al. Confiabilidade e pontuação mínima relacionada à intervenção analgésica de uma escala multidimensional para avaliação de dor pós-operatória em gatos. Arq Bras Med Veterinária e Zootec. 2013 Feb;65(1):153–62. Available from: http://www.scielo.br/scielo.php?script=sci_arttext&pid=S0102-09352013000100024&lng=pt&tlng=pt

17. Lorenz ND, Comerford EJ, Iff I. Long-term use of gabapentin for musculoskeletal disease and trauma in three cats. J Feline Med Surg. 2013;15(6):507–12. Available from: https://doi.org/10.1177/1098612X12470828

18. Rioja-Lang F, Bacon H, Connor M, Dwyer CM. Determining priority welfare issues for cats in the United Kingdom using expert consensus. Vet Rec Open. 2019;6(1):1–10.

19. Calvo G, Holden E, Reid J, Scott EM, Firth A, Bell A, et al. Development of a behaviour-based measurement tool with defined intervention level for assessing acute pain in cats. J Small Anim Pract. 2014 Dec;55(12):622–9. Available from: https://onlinelibrary.wiley.com/doi/10.1111/jsap.12280

20. Holden E, Calvo G, Collins M, Bell A, Reid J, Scott EM, et al. Evaluation of facial expression in acute pain in cats. J Small Anim Pract. 2014 Dec;55(12):615–21. Available from: https://onlinelibrary.wiley.com/doi/10.1111/jsap.12283

21. Frank D. Recognizing behavioral signs of pain and disease: A guide for practitioners. Vet Clin North Am - Small Anim Pract. 2014;44(3):507–24. Available from: http://dx.doi.org/10.1016/j.cvsm.2014.01.002

22. Seksel K. The Recognition and Assessment of Pain in Cats. Pain Manag Vet Pract. 2013;269–73.

23. Epstein M, Rodan I, Griffenhagen G, Kadrlik J, Petty M, Robertson S, et al. 2015 AAHA/AAFP pain management guidelines for dogs and cats. J Am Anim Hosp Assoc. 2015;51(2):67–84.

24. Ellis SLH. Recognising and assessing feline emotions during the consultation: History, body language and behaviour. J Feline Med Surg. 2018;20(5):445–56.

25. Horwitz DF, Rodan I. Behavioral awareness in the feline consultation: Understanding physical and emotional health. J Feline Med Surg. 2018;20(5):423–36.

26. Shipley H, Guedes A, Graham L, Goudie-DeAngelis E, Wendt-Hornickle E. Preliminary appraisal of the reliability and validity of the Colorado State University Feline Acute Pain Scale. J Feline Med Surg. 2019;21(4):335–9.

27. Hernandez-Avalos I, Mota-Rojas D, Mora-Medina P, Martínez-Burnes J, Casas Alvarado A, Verduzco-Mendoza A, et al. Review of different methods used for clinical recognition and assessment of pain in dogs and cats. Int J Vet Sci Med. 2019;7(1):43–54. Available from: https://doi.org/10.1080/23144599.2019.1680044

28. Hernández-Avalos I, Flores-Gasca E, Mota-Rojas D, Casas-Alvarado A, Miranda-Cortés AE, Domínguez-Oliva A. Neurobiology of anesthetic-surgical stress and induced behavioral changes in dogs and cats: A review. Vet World. 2021;14(2):393–404.

29. Gruen ME, Lascelles BDX, Colleran E, Gottlieb A, Johnson J, Lotsikas P, et al. 2022 AAHA Pain Management Guidelines for Dogs and Cats. J Am Anim Hosp Assoc. 2022;58(2):55–76.

30. Belli M, de Oliveira AR, de Lima MT, Trindade PHE, Steagall P V., Luna SPL. Clinical validation of the short and long UNESP-Botucatu scales for feline pain assessment. PeerJ. 2021 Apr 12;9:e11225. Available from: https://peerj.com/articles/11225

31. Evangelista MC, Watanabe R, Leung VSY, Monteiro BP, O’Toole E, Pang DSJ, et al. Facial expressions of pain in cats: the development and validation of a Feline Grimace Scale. Sci Rep. 2019;9(1):1–11.

32. Evangelista MC, Benito J, Monteiro BP, Watanabe R, Doodnaught GM, Pang DSJ, et al. Clinical applicability of the Feline Grimace Scale: Real-time versus image scoring and the influence of sedation and surgery. PeerJ. 2020;2020(4):1–17.

33. Bloor C, Allan L. Pain scoring systems in the canine and feline patient. Vet Nurse. 2017;8(5):252–8.

34. Stelow E. Behavior as an Illness Indicator. Vet Clin North Am Small Anim Pract [Internet]. 2020;50(4):695–706. Available from: https://linkinghub.elsevier.com/retrieve/pii/S019556162030019X

35. Mathews K, Kronen PW, Lascelles D, Nolan A, Robertson S, Steagall PV, et al. Guidelines for recognition, assessment and treatment of pain. Vet Nurse. 2015 Apr 2;6(3):164–73. Available from: https://onlinelibrary.wiley.com/doi/10.1111/jsap.12200

36. Merola I, Mills DS. Systematic review of the behavioural assessment of pain in cats. J Feline Med Surg. 2016;18(2):60–76.

37. Merola I, Mills DS. Behavioural Signs of Pain in Cats: An Expert Consensus. 2016; Available from: http://www.feline-friends.org.uk

38. Monteiro BP, Lascelles BDX, Murrell J, Robertson S, Steagall PVM, Wright B. 2022 WSAVA guidelines for the recognition, assessment and treatment of pain. J Small Anim Pract. 2022 Oct 27;1–79. Available from: https://onlinelibrary.wiley.com/doi/10.1111/jsap.13566

39. Pereira MAA, Campos KD, Evangelista MC, Gonçalves LA, Thurler RS, Nagashima JK, et al. Recognition and behavioral assessment of acute pain in cats: literature review. Brazilian J Vet Res Anim Sci. 2017;54(4):298.

40. Reid J, Scott EM, Calvo G, Nolan AM. Definitive Glasgow acute pain scale for cats: Validation and intervention level. Vet Rec. 2017 May;180(18):449. Available from: http://doi.wiley.com/10.1136/vr.104208

41. Robertson SA. Managing Pain in Feline Patients. Vet Clin North Am - Small Anim Pract. 2008;38(6):1267–90.

42. Robertson S. How do we know they hurt? Assessing acute pain in cats. In Pract [Internet]. 2018 Dec 29;40(10):440–8. Available from: https://onlinelibrary.wiley.com/doi/10.1136/inp.k4158

43. Robertson S. Feline acute pain series: assessment of acute pain in cats. Today’s Vet Pract. 2014;4(1):33-36,38. Available from: http://todaysveterinarypractice.epubxp.com/i/255349

44. Steagall P V. Analgesia: What Makes Cats Different/Challenging and What Is Critical for Cats? Vet Clin North Am - Small Anim Pract. 2020;50(4):749–67.

45. Steagall P V, Robertson S, Simon B, Warne LN, Shilo-Benjamini Y, Taylor S. 2022 ISFM Consensus Guidelines on the Management of Acute Pain in Cats. J Feline Med Surg. 2022;24(1):4–30.

46. Steagall P V., Monteiro BP. Acute pain in cats: Recent advances in clinical assessment. J Feline Med Surg. 2019;21(1):25–34.

47. Waran, N., Best, L., Williams V, Waran N, Best L, Williams V, Salinsky J, Dale A, et al. A preliminary study of behaviour-based indicators of pain in cats. Animal Welfare. Anim Welf. 2007;16(SUPPL.):105–8.

48. Watanabe R, Frank D, Steagall P V. Pain behaviors before and after treatment of oral disease in cats using video assessment: A prospective, blinded, randomized clinical trial. BMC Vet Res. 2020;16(1):1–11.

49. Association of Veterinary Teachers and Research Workers. Guidelines for the recognition and assessment of pain in cats. Veterinary Record (1986) 118, 334-338.

50. Hellyer, P.; Gayner., J. Acute postsurgical pain in cats and dogs. The compendium on continuing education for the practicing veterinarian. Colorado State University. 20:2140-153 (1998).

51. Barrat, L. Feline pain assessment and scoring systems. The Veterinary Nurse. 2013;4(8):470-477

52. Berry, S. Analgesia in the perioperative period. Vet Clin North Am Small Pract. 2015;25(5)1013-27.

53. White, K. Assessment of acute pain in cats: signs, tools and limitations. Companion Animal. 2016;21(8)
